# TI17, a novel compound, exerts anti‐MM activity by impairing Trip13 function of DSBs repair and enhancing DNA damage

**DOI:** 10.1002/cam4.6706

**Published:** 2023-11-09

**Authors:** Shuaikang Chang, Wenqin Xiao, Yongsheng Xie, Zhijian Xu, Bo Li, Guanli Wang, Ke Hu, Yong Zhang, Jinfeng Zhou, Dongliang Song, Huabin Zhu, Xiaosong Wu, Yumeng Lu, Jumei Shi, Weiliang Zhu

**Affiliations:** ^1^ Department of Hematology, Shanghai East Hospital Tongji University School of Medicine Shanghai China; ^2^ Department of Gastroenterology, Shanghai General Hospital Shanghai Jiao Tong University School of Medicine Shanghai China; ^3^ Department of Hematology, Shanghai Tenth People's Hospital Tongji University School of Medicine Shanghai China; ^4^ State Key Laboratory of Drug Research, Drug Discovery and Design Center, Shanghai Institute of Materia Medica Chinese Academy of Sciences Shanghai China

**Keywords:** DNA damage, DSBs repair, myeloma, TI17, Trip13

## Abstract

**Background:**

Thyroid hormone receptor interacting protein 13 (Trip13) is an AAA‐ATPase that regulates the assembly or disassembly protein complexes and mediates Double‐strand breaks (DSBs) repair. Overexpression of Trip13 has been detected in many cancers and is associated with myeloma progression, disease relapse and poor prognosis inmultiple myeloma (MM).

**Methods:**

We have identified a small molecular, TI17, through a parallel compound‐centric approach, which specifically targets Trip13. To identify whether TI17 targeted Trip13, pull‐down and nuclear magnetic resonance spectroscopy (NMR) assays were performed. Cell counting kit‐8, clone formation, apoptosis and cell cycle assays were applied to investigate the effects of TI17. We also utilized a mouse model to investigate the effects of TI17 in vivo.

**Results:**

TI17 effectively inhibited the proliferation of MM cells, and induced the cycle arrest and apoptosis of MM cells. Furthermore, treatment with TI17 abrogates tumor growth and has no apparent side effects in mouse xenograft models. TI17 specifically impaired Trip13 function of DSBs repair and enhanced DNA damage responses in MM. Combining with melphalan or HDAC inhibitor panobinostat triggers synergistic anti‐MM effect.

**Conclusions:**

Our study suggests that TI17 could be acted as a specific inhibitor of Trip13 and supports a preclinical proof of concept for therapeutic targeting of Trip13 in MM.

## Graphical abstract

TI17 specifically impaired Trip13 function of DSBs repair and enhanced DNA damage responses in MM cells.

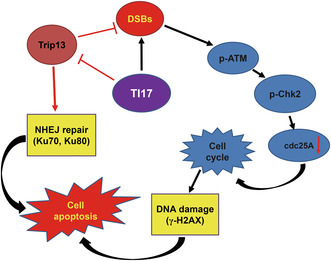

## 1 INTRODUCTION

Multiple myeloma (MM) is a malignant monoclonal tumor characterized by its prominent clonal proliferation of plasma cells in the bone marrow, which accounts for about 1% of neoplastic diseases and 10% of hematologic malignancies.^1^ Although the improvement of MM in novel therapies, the prognosis of MM remains poor, especially in the relapsed patients.^2^, ^3^, ^4^ In addition, most MM patients will occur disease recurrence after primary treatment.^5^ Therefore, scientists have been trying to develop effective and safe drugs for many years.

Double‐strand breaks (DSBs), the most cytotoxic types of DNA damage, are difficult to repair, and can lead to genome rearrangements and cell death.^6^, ^7^ And nonhomologous end joining (NHEJ)^8^ is the major pathway for the efficient repair of DSBs, since it can occur throughout the cell cycle and is favored in G1 cells.^9^ Thyroid hormone receptor interacting protein 13 (Trip13, or HPV16E1BP), an AAA‐ATPase, is the mouse ortholog of pachytene checkpoint 2 (Pch2).^10^, ^11^, ^12^ Trip13 is expressed in multiple somatic tissues.^13^, ^14^ Meanwhile, overexpression of Trip13 may promote the development of cancer.^15^ Increasing evidence has suggested that Trip13 is necessary for recombination repair of meiotic DSBs in mice.^16^, ^17^, ^18^ In prostate cancer, Trip13 promotes the early repair of DSBs, and its overexpression is associated with poor prognosis.^19^ In head and neck cancer, Trip13 could promote error‐prone NHEJ, enhance the repair of DSBs, and induce treatment resistance.^20^ These findings suggest that Trip13 protein can serve as a kind of important monitoring, through the provision of DNA repair mechanisms to help assess the DNA integrity.

Trip13 is a chromosomal instability gene in MM, which is related to drug resistance and disease relapse,^21^ and it has also been considered to be an important highly expression gene in a 70‐gene model of high‐risk myeloma by extensive gene expression profiling.^22^ In a previous study, we have found that Trip13 is significantly up‐regulate at relapse MM patients after initial chemotherapy.^23^

Here, this study details our efforts in identifying a novel small molecular inhibitor, TI17, which was identified from a compound library and confirmed to bind to Trip13. Then we further evaluated the potential anti‐MM activity of TI17 in MM cells by impairing the Trip13 function of DSBs repair in vitro and in vivo.

## 2 MATERIALS AND METHODS

### 2.1 Cell lines and culture

U266 and BM stroma cell HS‐5 were obtained from the American Type Culture Collection (ATCC) (Manassas, VA, USA). ARP‐1, OCI‐MY5, ARK, KMS11, RPMI‐8226 (bortezomib‐sensitive) and RPMI‐8226/R5 (bortezomib‐resistant) were kindly gift from Professor Fenghuang Zhan (Department of Internal Medicine, University of Iowa, Iowa City, IA, USA). H929S (bortezomib‐sensitive) and H929R (bortezomib‐resistant) were kindly obtained from Professor Jian Hou (Department of Hematology, Changzheng Hospital, The second military Medical University, Shanghai, China). Human lung cancer cell A549, prostate cancer cell PC‐3, breast cancer cell MDA‐MB‐231, nasopharyngeal cancer cell 5‐8F, liver cancer cell LM3, and renal cancer cell A498 were kindly provided by others research group in my lab.

All MM cell lines and A549, PC‐3, 5‐8F cells were maintained in RPMI‐1640 medium (Gibco, BRL, USA) supplemented with 10% fetal bovine serum (FBS; Gibco, BRL, USA) and 1% penicillin–streptomycin (PS; Gibco, BRL, USA). Human HS‐5, MDA‐MB‐231, LM3, and A498 cells were cultured in DMEM/high glucose medium (Gibco, BRL, USA) containing 10% FBS and 1% PS. All cells were maintained in a humidified atmosphere of 5% CO_2_ at 37°C. Culture medium was changed every other day. Primary cells assay was performed as described previously.^24^ Informed consent was obtained from each patient and healthy donor. The protocol for collection and usage of clinical samples was approved by the institutional review board of Shanghai Tenth People's Hospital, Shanghai, China. Informed consent was obtained in accordance with the Declaration of Helsinki.

### 2.2 Reagents

TI17 was dissolved in dimethyl sulfoxide (DMSO; Sigma, St Louis, MO, USA) and stored at −20°C until use. HDAC inhibitor panobinostat, doxorubicin (DOX) and melphalan (MEL) were obtained from Sigma (S. Louis, MO, USA). Recombinant human interleukin‐6 (IL‐6) was purchased from Biosource, CA, USA. Recombinant human insulin growth factor‐1 (IGF‐1) was obtained from R&D Systems (Minneapolis, MN, USA). Pan‐caspase inhibitor Z‐VAD‐FMK was obtained from Selleckchem (Houston, TX, USA).

### 2.3 Cell viability assay

Cell viability assay was performed according to a previous study.^24^ Briefly, cells were seeded into 96‐well plates and then treated with TI17. Cell viability was determined using the Cell Counting Kit (CCK)‐8 assays.

### 2.4 Clonogenic assay

MM cell lines were inoculated in 12‐well plates at 1000 cells per well and cultured at 37°C for 2 weeks. Cell colonies with 0.1% crystal violet staining for 30 min. Colonies with at least 50 cells were counted.

### 2.5 EdU assay

MM cells were treated with TI17 (0, 5, and 10 μM) for 24 h, and cells were collected. Incorporation of 5‐ethynyl‐2′‐deoxyuridine (EdU) was determined using an EdU kit (RiboBio, Guangzhou, China) according to the instruction.

### 2.6 Cell cycle analysis

Cell viability assay was determined with reference to previous study.^24^ Briefly, cells were exposed with TI17. Then cells were harvested, with propidium iodide (PI) (BD Pharmingen, Franklin Lakes, NJ, USA) incubation, and then analyzed by flow cytometry.

### 2.7 Apoptosis analysis

Apoptosis assay was performed according to a previous study.^24^ Briefly, cells were treated with or without TI17. Then, cells were collected and stained, and then analyzed by flow cytometry.

### 2.8 Immunofluorescence staining

Immunofluorescence staining assay was determined with reference to previous study.^24^ Briefly, Cells were incubated with Ki‐67 (1:200, dilution, Abcam, Cambridge, MA, USA) and γ‐H2AX (1:200, dilution, Abcam, Cambridge, MA, USA) in the incubator at 37°C for 1 h.

### 2.9 Western blot analysis

Western blot assay was performed according to a previous study.^24^ Primary antibodies against various proteins were as follows: anti‐cleaved caspase‐3, anti‐cleaved caspase‐8, anti‐cleaved caspase‐9, anti‐BCL2, anti‐BCL‐XL, anti‐BAX were obtained from Cell Signaling Technology (CST, Beverly, USA); anti‐CDK4, anti‐CDK6, anti‐CyclinD1, anti‐Trip13, anti‐Ku70, anti‐Ku80, anti‐H2AX, anti‐phospho‐ H2AX (γ‐H2AX), anti‐CHK2, anti‐phospho‐CHK2 (Thr68), anti‐ATM, anti‐phospho‐ATM (Ser1981), and anti‐GAPDH were obtained from Abcam (Cambridge, MA, USA); anti‐β‐actin was obtained from Sigma (S. Louis, MO, USA).

### 2.10 Pull‐down analysis

Pull‐down assay was determined with reference to previous study.^25^ Briefly, the cell lysate was then incubated with 50 μmol/L TI17‐biotin or biotin in the presence of neutral fat sugar resins, and analyzed by immunoblotting.

### 2.11 Tumor xenograft models

Tumor xenograft models were performed as described previously.^24^ Briefly, 3 × 10^6^ OCI‐MY5 cells were injected subcutaneously into the upper flank region of the BALB/C nude mice. Then mice were randomly divided into three groups: the control group, TI17(50 mg/kg) and TI17 (100 mg/kg).

### 2.12 TUNEL assay

TUNEL assay were performed as described previously.^24^ Cell apoptosis was evaluated by use a light microscope by three pathologists who do not know the original specimens.

### 2.13 Statistical analysis

Results were presented as mean ± standard deviation (SD). SPSS v20.0 software (IBM, Armonk, NY, USA) was used for Student's *t*‐test and one‐way analysis of variance (ANOVA). *p* < 0.05 was considered statistically significant.

## 3 RESULTS

### 3.1 TI17 directly targeted Trip13

**FIGURE 1:**
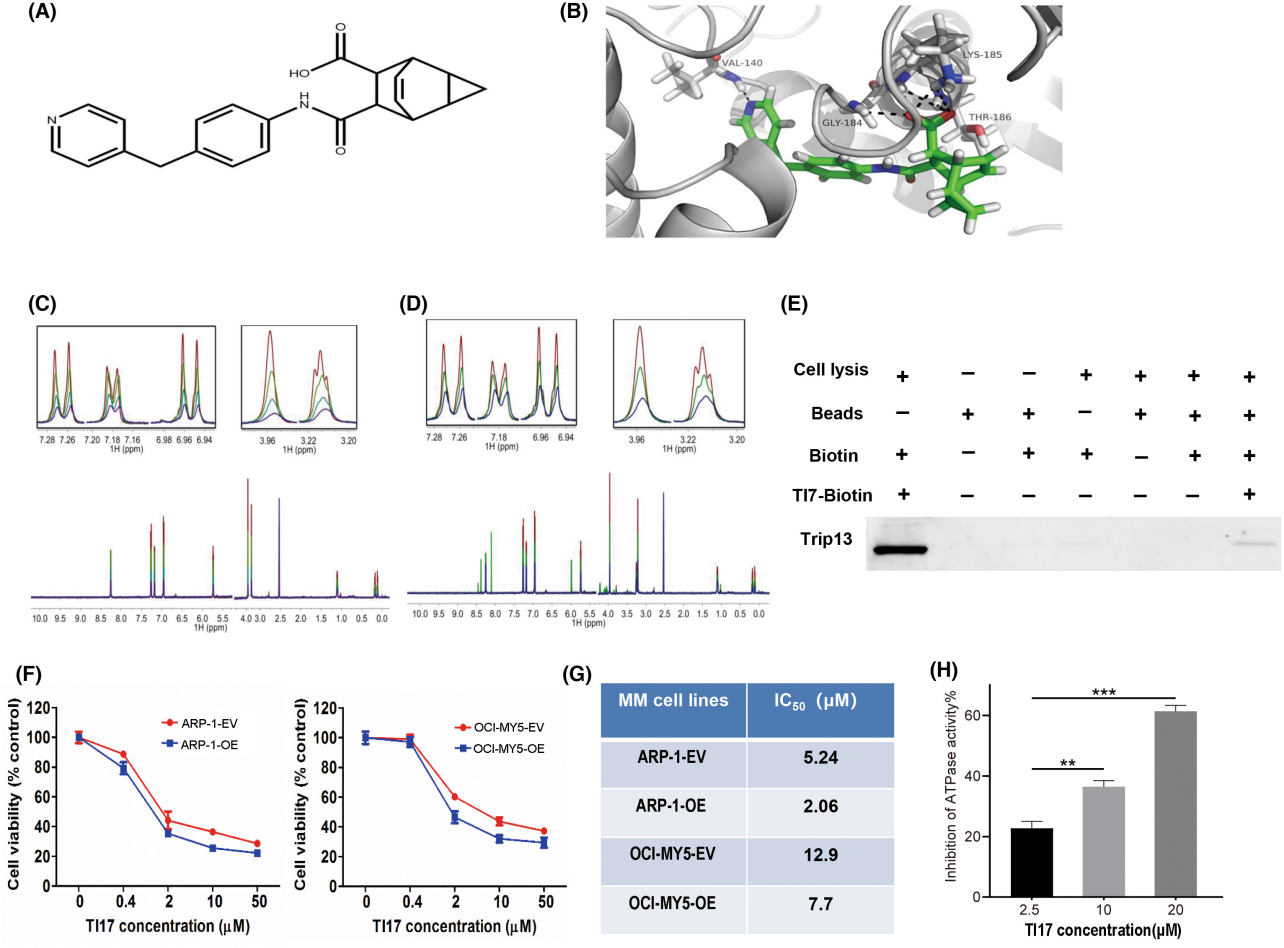
TI17 directly targeted Trip13. (A) Chemical structure of TI17. (B) Molecular docking results of TI17 and TRIP13 protein. (C) T1ρ NMR spectra are displayed for 200 μM TI17 alone (red), and at 40:1 (green), 20:1 (cyan) and 10:1 (blue) TI17:Trip13 molar ratio. (D) T1ρ NMR spectra are displayed for 200 μM TI17 alone (red), 200 μM TI17 in the presence of 10 μM Trip13 (blue), 200 μM TI17 in the presence of 10 μM Trip13 and 200 μM ADP (green). (E) Affinity‐pulldown by using TI17‐conjucted matrix or control matrix to incubate with ARP‐1 cells extract, lanes 7, 8 are the matrix bound proteins were competed with TI17, and resultant precipitates were detected by using western blot. (F) Trip13‐OE ARP‐1 or OCI‐MY5 cells, and empty vector‐transfected cells were treated with DMSO or with TI17 for 48 h and cell viability was measured by CCK8 kit. (G) IC_50_ of these cells after DMSO or TI17 treatment was determined by using CalcuSyn software, Version 2.1. (H) Relative ATPase activity was examined after TI17 treatment by ADP‐Glo™ Kinase Assay. All results are expressed as mean ± SD of three independent experiments and **p ≤ 0.01, ***p ≤ 0.001.

Based on our previous research, we knew that Trip13 involved in the development and prognosis of MM. To search for such effective compounds, we first screened a compound library, and chose only one named TI17 (Figure 1A,B) for the further research. We first elucidated the binding mode of TI17 to Trip13 by using the ligand observed T1ρ nuclear magnetic resonance (NMR) analysis. Dose‐dependent effects observed in the T1ρ titration experiments of Trip13 to TI17 indicate that the binding between these two molecules is specific (Figure 1C). Additionally, T1ρ titration data suggest a mutually exclusive binding of TI17 and ADP to Trip13 (Figure 1D). Meanwhile, we detected an affinity‐pulldown target verification system, in which TI17 was coupled to a biotin via a multi‐step synthesis. Comparing with the control matrix, mixing the TI17‐affinity biotin with the cell lysate reduced the endogenous Trip13 (Figure 1E). We also found that TI17 inhibited the growth of MM cells in a dose‐dependent manner, while overexpression of TRIP13 increased sensitivity to TI17 as detected by CCK8 kit assays (Figure 1F,G). In further, we examined whether TI17 inhibited the ATPase activity of TRIP13. These data showed that the ATPase activity of TRIP13 was inhibited by TI17 in a dose‐dependent manner (Figure 1H). These experimental results indicate that TI17 directly target Trip13.

### 3.2 TI17 inhibits proliferation of MM cells and overcomes the protective effect of the BM environment on MM cells in vitro

**FIGURE 2:**
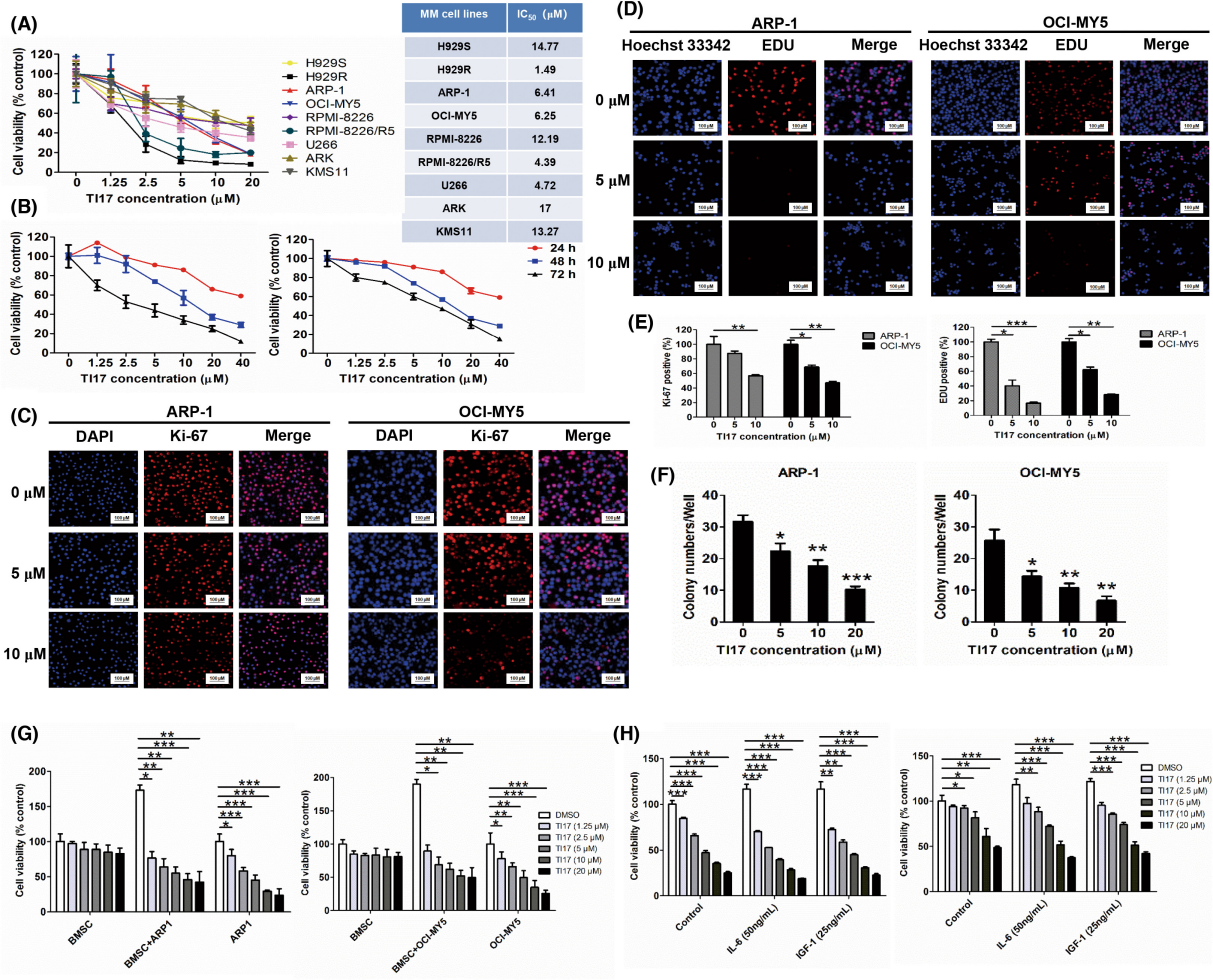
TI17 inhibited proliferation of MM cells and overcame the protective effect of the BM environment on MM cells in vitro. (A) MM cell lines were treated with DMSO or with TI17 for 48 h and cell viability was measured by CCK8 kit. (B) ARP‐1 (left panel) and OCI‐MY5 (right panel) cells were treated with DMSO or with TI17 for 24, 48, and 72 h, and analyzed for cell viability by CCK8 kit. Immunofluorescence staining of Ki‐67 (C) and EDU (D) in cells after DMSO or TI17 treatment for 24 h. Red indicate Ki‐67 or EDU positive cells. 250× magnification. (E) Analysis of Ki‐67 and EDU staining. (F) Cells were treated with DMSO or with TI17 for 14 days, and colony numbers were quantified with software ImageJ. 50× magnification. (G) Cells were cultured with or without BMSC in the presence or absence of TI17 for 48 h and analyzed for cell viability by CCK8 kit. (H) ARP‐1 (Left panel) and OCI‐MY5 (Right panel) cells were cultured with or without IL‐6 and IGF‐1 in the presence or absence of TI17 for 48 h and analyzed for cell viability by CCK8 kit. All results are expressed as mean ± SD of three independent experiments and *p ≤ 0.05, **p ≤ 0.01, ***p ≤ 0.001.

In this study, we further evaluated the expression of Trip13 by western blot in human MM cells, our findings shown that the protein level of Trip13 was elevated in MM cells (Figure S1). And our data demonstrated that the viability of these MM cells was obviously inhibited by TI17 treatment (Figure 2A). IC_50_ of TI17 at concentrations of 1.25, 2.5, 5, 10, and 20 μM was detected in each these MM cells for inhibition of cell viability using CalcuSyn software, Version 2.1 (Figure 2A). Interestingly, we also found TI17 induced a time‐ and dose‐dependent decline in the viability of ARP1 and OCI‐MY5 cells (Figure 2B).

Importantly, as in Figure S2A, we observed the level of Trip13 was increased in other tumor cells, which is consistent with previous studies in these cancers.^13^, ^19^, ^20^, ^26^, ^27^ Meanwhile, results presented that the activity of these cancer cells was slightly inhibited after TI17 intervention, compared to MM cells (Figure S2B,C). These data showed that TI17 also has anti‐proliferation effect on other cancer cells, although this effect was less sensitive than on MM cell lines.

We found that the expression of Ki‐67 was significantly decreased after TI17 intervention (Figure 2C,E). Furthermore, EDU incorporation assay demonstrated that TI17 significantly decreased DNA synthesis in ARP‐1 and OCI‐MY5 cells for 24 h (Figure 2D,E). In addition, the results of colonogenic survival assays further confirmed the anti‐proliferation activity of TI17 towards at least two MM cells (ARP‐1 and OCI‐MY5) (Figure 2F). Taken together, these results collectively indicated that TI17 significantly inhibits the proliferation of MM cells.

ARP1 and OCI‐MY5 cells were cultured in the presence or absence of BMSC, IL‐6 and IGF‐1 at various concentrations of TI17 for 48 h. Results showed that TI17 treatment significantly inhibits the cell viability of ARP1 and OCI‐MY5 cells when BMSC were present (Figure 2G), as well as IL‐6 and IGF‐1 (Figure 2H), while TI17 treatment has no significant effect on the viability of BMSCs. These results indicated that TI17 can not only directly impact on MM cells, but also overcome the protective effect of the BM microenvironment on MM cells.

### 3.3 TI17 induced G_0_ /G_1_ arrest and apoptosis in MM cells in vitro

**FIGURE 3:**
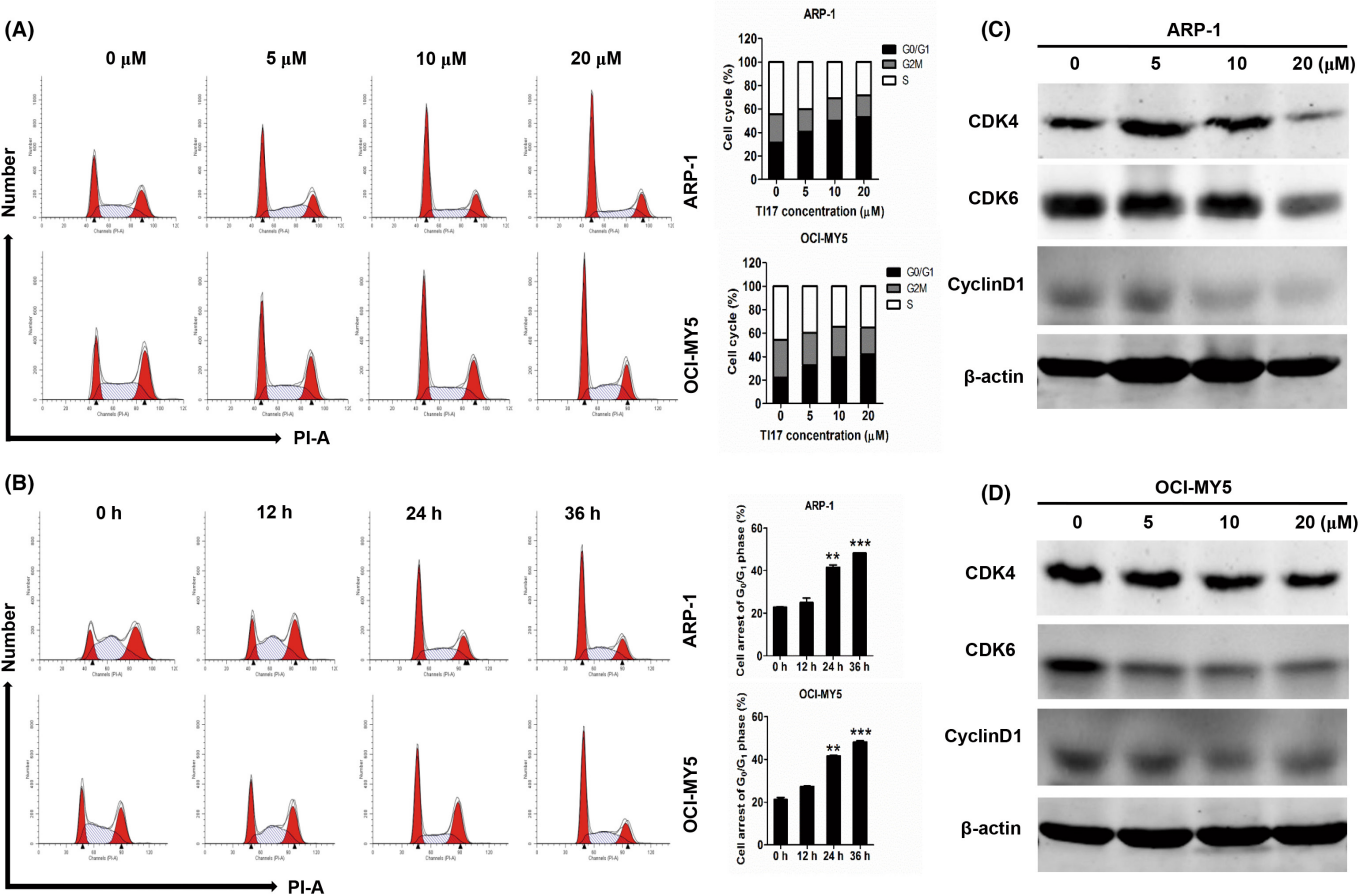
TI17 induced G_0_/G_1_ arrest in MM cells in vitro. (A, B) Cell‐cycle analysis of TI17‐treated cells. (C, D) Western blot analysis for CDK4, CDK6, cyclin D in cells after DMSO or TI17 treatment for 24 h. All results are expressed as mean ± SD of three independent experiments and **p ≤ 0.01, ***p ≤ 0.001.

**FIGURE 4:**
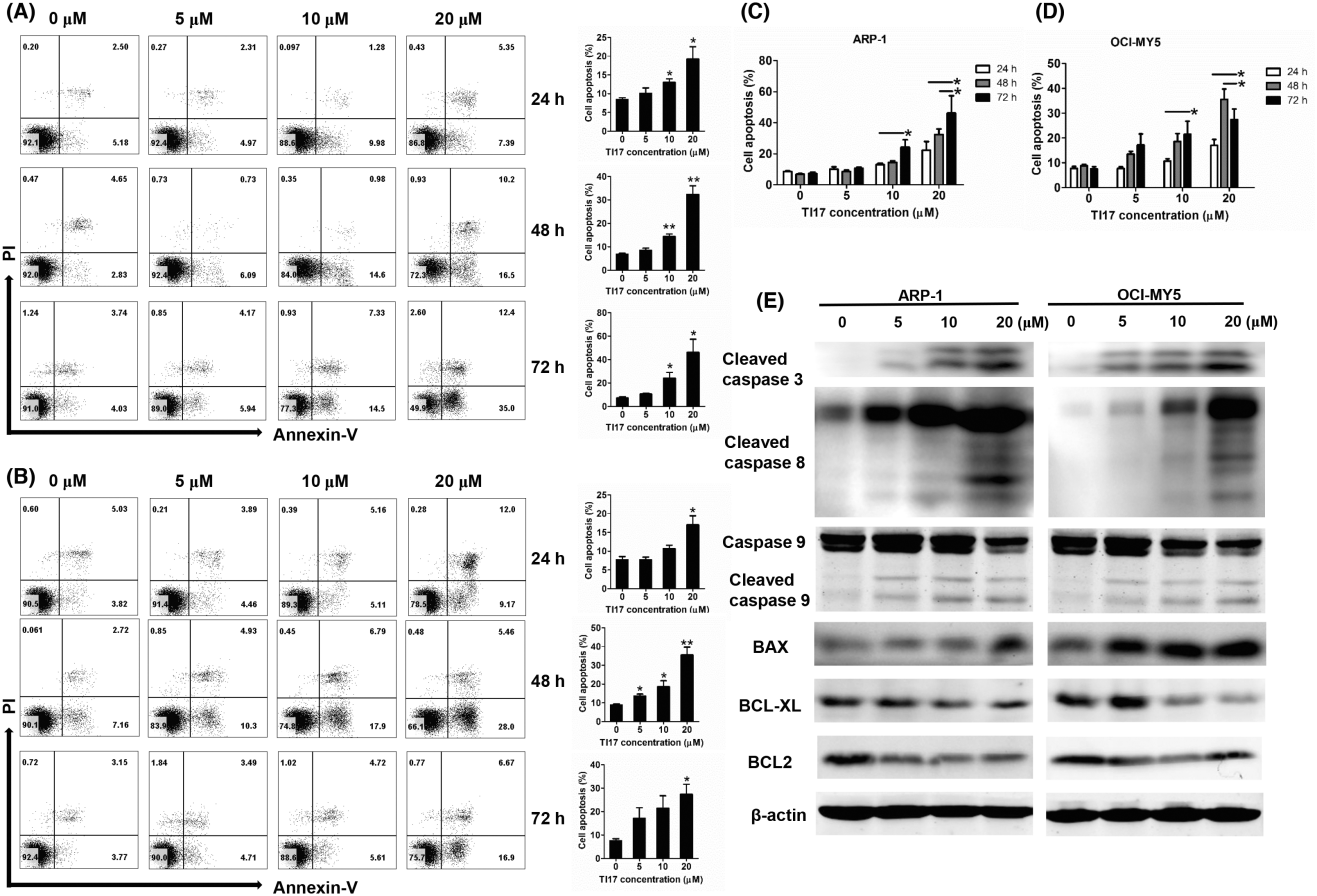
TI17 induced apoptosis in MM cells in vitro (A‐D) Cells were treated with DMSO or with TI17 for 24, 48, 72 h, and measured the cell apoptosis by Annexin V/PI double staining using flow cytometry. (E) Western blot analysis for cleaved‐caspase 3, cleaved‐caspase 8, cleaved‐caspase 9, Bcl‐2, Bcl‐xl, and BAX in cells after DMSO or TI17 treatment for 48 h. All results are expressed as mean ± SD of three independent experiments and *p ≤ 0.05, **p ≤ 0.01.

**FIGURE 5:**
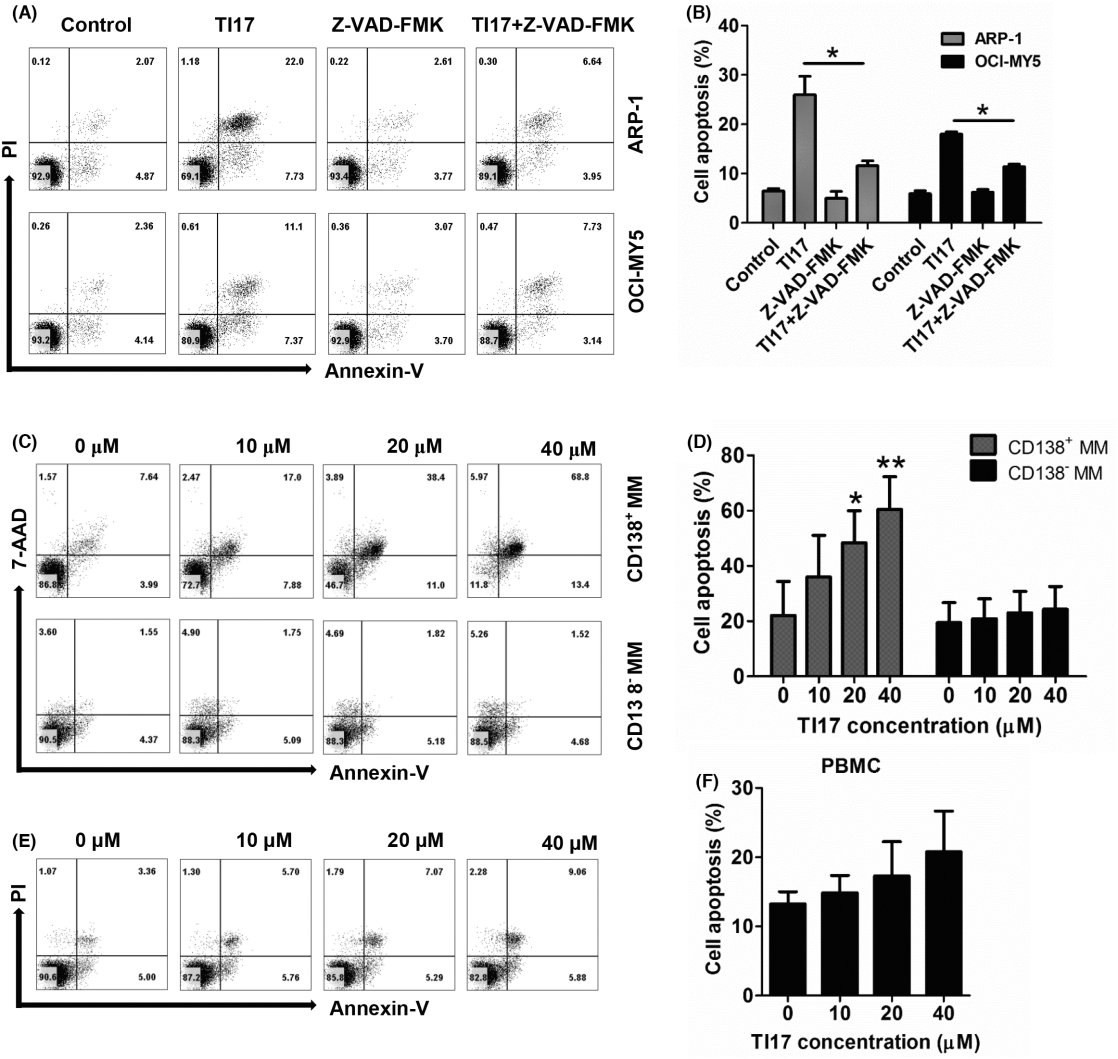
TI17‐triggered apoptosis mediated by caspases and TI17 induces patient MM cells apoptosis. (A, B) Cells were pretreated with pan‐caspase (Z‐VAD‐FMK) for 2 h, then added DMSO or TI17 (10 μM) for another 48 h, and measured the cell apoptosis by Annexin V/PI double staining using flow cytometry. (C, D) Primary patient MM cells (CD138^+^, CD138^−^) were treated with DMSO or TI17 for 48 h, and detected the cell apoptosis by anti‐CD138/Annexin‐V/7‐AAD staining using flow cytometry. (E, F) Normal PBMCs were treated with DMSO or with TI17 for 48 h, and measured the cell apoptosis by Annexin V/PI double staining using flow cytometry. All results are expressed as mean ± SD of three independent experiments and *p ≤ 0.05, **p ≤ 0.01.

We next examined the effects of TI17 on the cell cycle in MM cells in vitro by flow cytometry analysis. As shown in Figure 3A,B, treatment of TI17 induced obvious cell accumulation of G_0_/G_1_ DNA content in ARP1 and OCI‐MY5 cells. Meanwhile, the expression levels of CDK4, CDK6, and cyclinD1 were markedly downregulated after TI17 treatment (Figure 3C,D), further conforming that TI17 could significantly induce cell cycle arrest. Furthermore, As presented in Figure 4A–D, administration of TI17 also caused significant increase in the rate of apoptosis in a concentration‐ and time‐dependent manner in ARP‐1 and OCI‐MY5 cells, as well as in H929R and H929S (supplementary Figure S3A,B). Meanwhile, western blot analysis indicated that the expression levels of cleavage caspase‐3, cleavage caspase‐8, cleavage caspase9, as well as BAX were upregulated, while BCL‐XL and BCL2 were down‐regulated in ARP‐1 and OCI‐MY5 cells after TI17 intervention (Figure 4E). Meanwhile, we found that pan‐caspase inhibitor Z‐VAD‐FMK significantly eliminated TI17‐induced apoptosis of ARP‐1 and OCI‐MY5 cells (Figure 5A,B). These results indicated that TI17 triggers both exogenous and endogenous apoptotic pathways, and MM cells apoptosis induced by TI17 is mediated via caspases.

In order to determine whether TI17 has anti‐MM activity on patient MM cells, we detected the effect of TI17 on patients CD138^+^ MM cells by flow cytometry analysis, and our findings showed that TI17 induced a significant dose‐dependent increase apoptosis in primary CD138^+^ MM cells, while have no cytotoxic effect on CD138^−^ MM cells (Figure 5C,D), as well as normal PBMCs (Figure 5E,F), indicating TI17 is a favorable therapeutic index in MM.

### 3.4 TI17 impaired DSBs repair and enhanced DNA damage responses in MM cells in vitro

**FIGURE 6:**
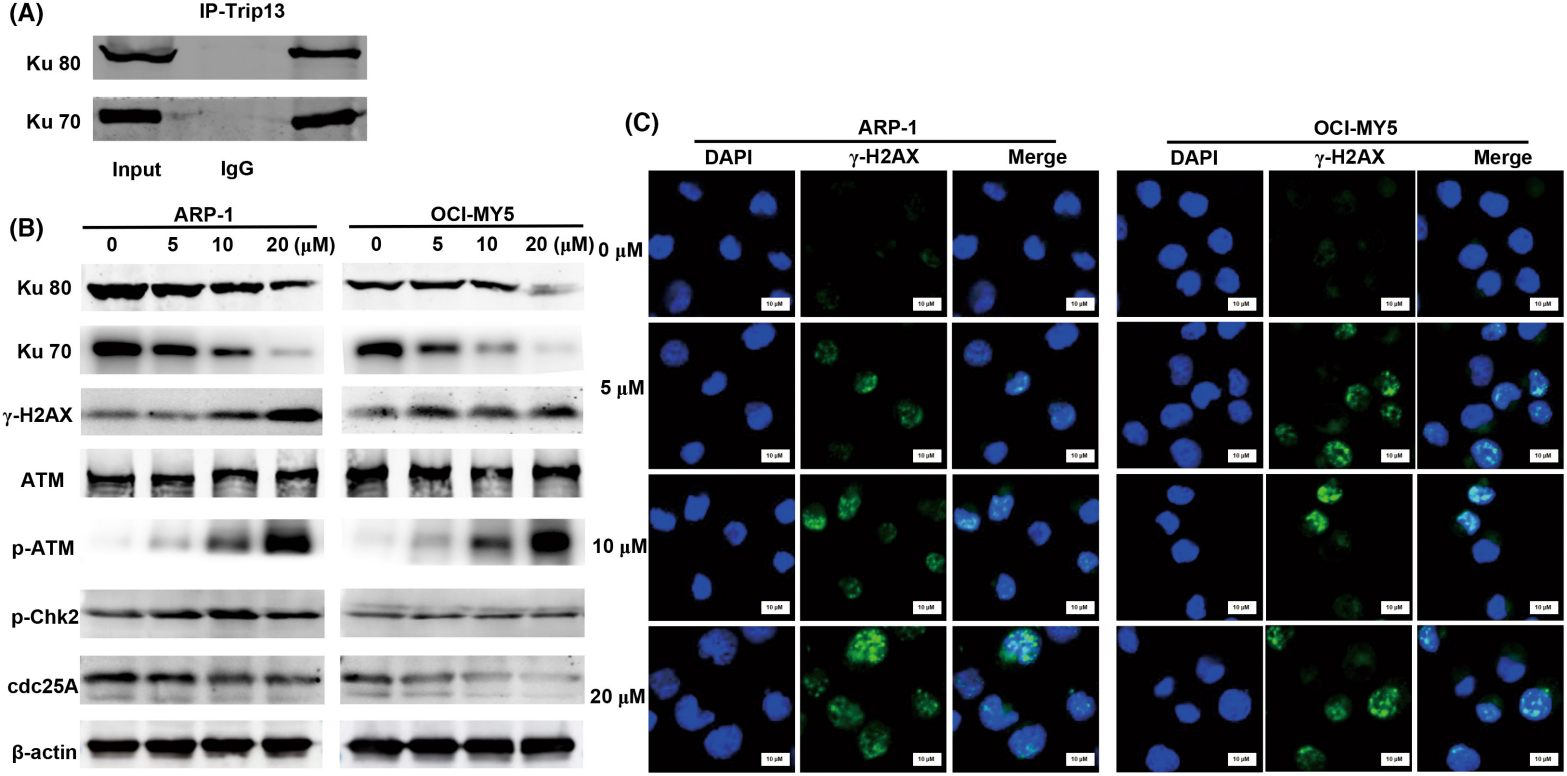
TI17 enhanced DNA damage in MM cells in vitro. (A) Trip13 was immunoprecipitated in the presence or absence of ethidium bromide and immunoblotted with anti‐Ku70, anti‐Ku80 and anti‐Trip13 antibodies in ARP‐1 cells. (B) Western blot analysis for Ku70, Ku80, γ‐H2AX, ATM, p‐ATM, Chk2, p‐Chk2, and cdc25A in cells after TI17 treatment for 48 h. (C) Immunofluorescence staining of γ‐H2AX in cells after TI17 treatment for 24 h. Green indicate γ‐H2AX positive cells. 1260× magnification.

Then we further performed whether Trip13 could interact with Ku70 and Ku80 proteins in MM cell lines by co‐immunoprecipitated assay. As shown in Figure 6A, Trip13 could co‐immunoprecipitate with Ku70 and Ku80 proteins but not control IgG in ARP‐1 cells. Second, we observed that TI17, which can target Trip13, could inhibit the repair of NHEJ by decreasing the expression of Ku70 and Ku80 via using western blot analysis in ARP‐1 cells (Figure 6B).

H2AX plays an important role in the DNA damage response and is phosphorylated at Ser‐139 (γ‐H2AX) in response to DSBs.^28^ Therefore, to assess the effect of TI17 on DSB‐repair in MM cell lines, we investigated the expression of γ‐H2AX by western blot and immunofluorescence analysis in ARP‐1 and OCI‐MY5 cells (Figure 6B). As shown in Figure 6C, TI17 markedly increased the expression of γ‐H2AX in ARP‐1 and OCI‐MY5 cells.

Moreover, ataxia telangiectasia mutated (ATM), one of the phosphoinositide (PI) 3‐like kinases,^29^ which is triggered by DSBs can phosphorylate various downstream substrates such as H2AX and cell cycle checkpoint kinases Chk2.^30^ Therefore, we next detected the expression of phosphorylated (p)‐ATM, as well as its downstream effector p‐Chk2. Results showed that TI17 increased the levels of p‐ATM and p‐Chk2, while decreased the level of cdc25A (Figure 6B).

### 3.5 In vivo anti‐MM activity of TI17

**FIGURE 7:**
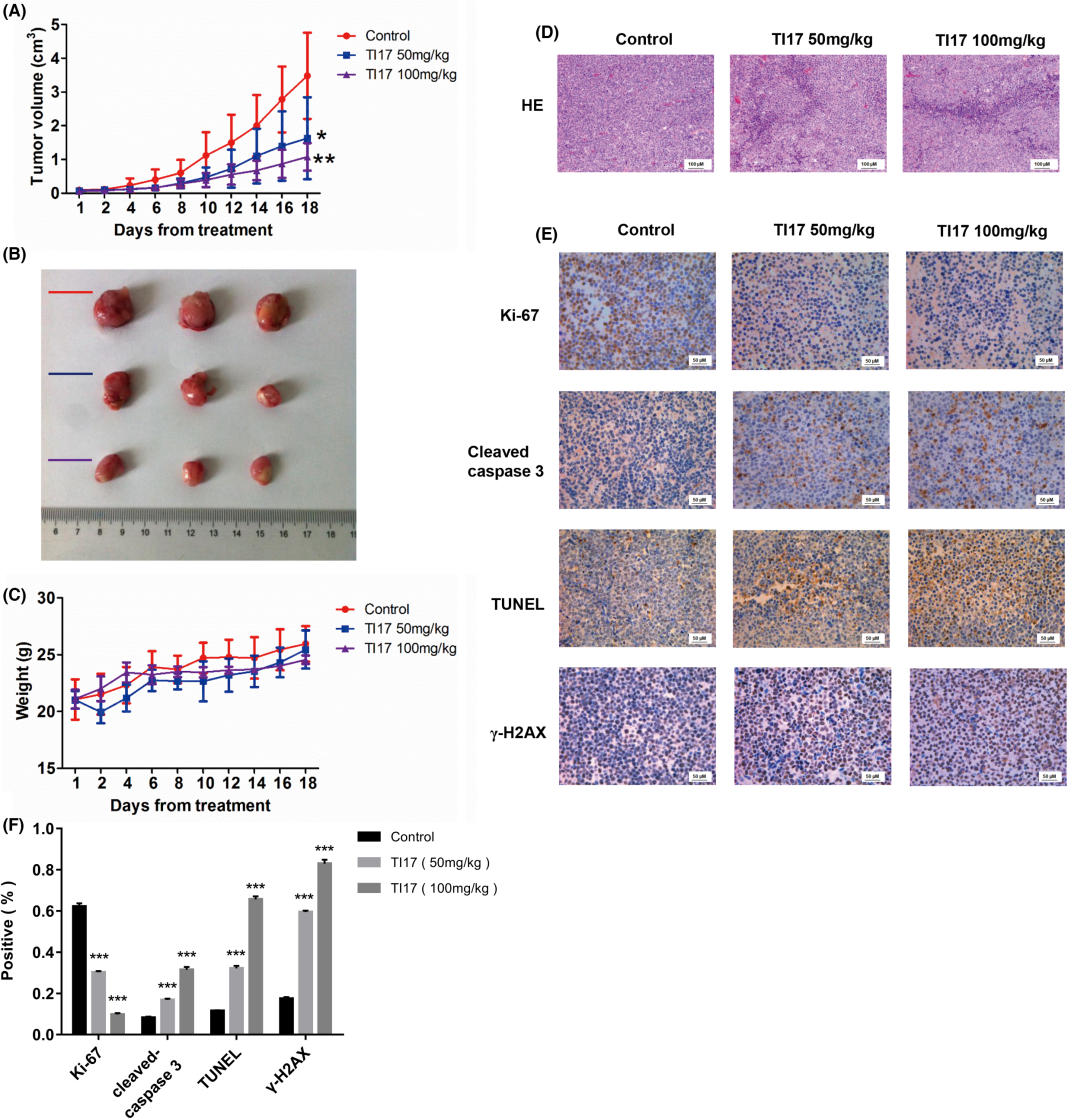
In vivo anti‐MM activity of TI17. (A) OCI‐MY5 cells were injected subcutaneously into mice and mice were treated with vehicle, 50 mg/kg, and 100 mg/kg TI17 every day for 18 days via intraperitoneal injection after tumors formed within 2 weeks. Tumor size was measured every other day. Five mice/group. (B) Gross appearance of tumors. (C) Mice body weight was measured every other day. (D) HE staining of tumor sections for detected the tumor histological after TI17 treatment. 200× magnification. (E, F) Immunohistochemical staining of Ki‐67, cleaved‐caspase 3, TUNEL, and γ‐H2AX in vivo after TI17 treatment. 400× magnification. All results are expressed as mean ± SD of three independent experiments and *p ≤ 0.05, **p ≤ 0.01, ***p ≤ 0.001.

In order to determine the anti‐MM efficacy of TI17 in vivo, we subsequently examined the tumor inhibition activity in a MM xenograft murine model. We gave TI17 or vehicles daily to mice intravenously. As seen in Figure 7A,B, administration of TI17 significantly reduced the growth of tumor in a concentration‐dependent manner (50 mg/kg, *p* = 0.048; 100 mg/kg, *p* = 0.0039), compared with vehicle‐treated animals. Importantly, there were no significant difference in body weight among the groups (Figure 7C). Furthermore, HE staining indicated that TI17 induced a significant increase in cell shrinkage and fragmentation of harvested tumors (Figure 7D). Meanwhile, results showed that no significant histological changes in heart, liver, lung, and kidney in all mice (Figure S4), indicating that TI17 had minimal side effects. As shown in Figure 7E,F, TI17 significantly decreased the tumor proliferation by decreasing Ki‐67 expression and induced tumor apoptosis by increasing the number of cleaved‐caspase 3‐ and TUNEL‐positive cells. Meanwhile, our results showed that TI17 could significantly enhanced DNA damage by increasing the expression of γ‐H2AX, which was consistent with our results in vitro. These results showed that TI17 has potent activity in inhibiting tumor growth in animals.

### 3.6 TI17 effects on MM cells in combination with melphalan or HDAC inhibitor

**FIGURE 8:**
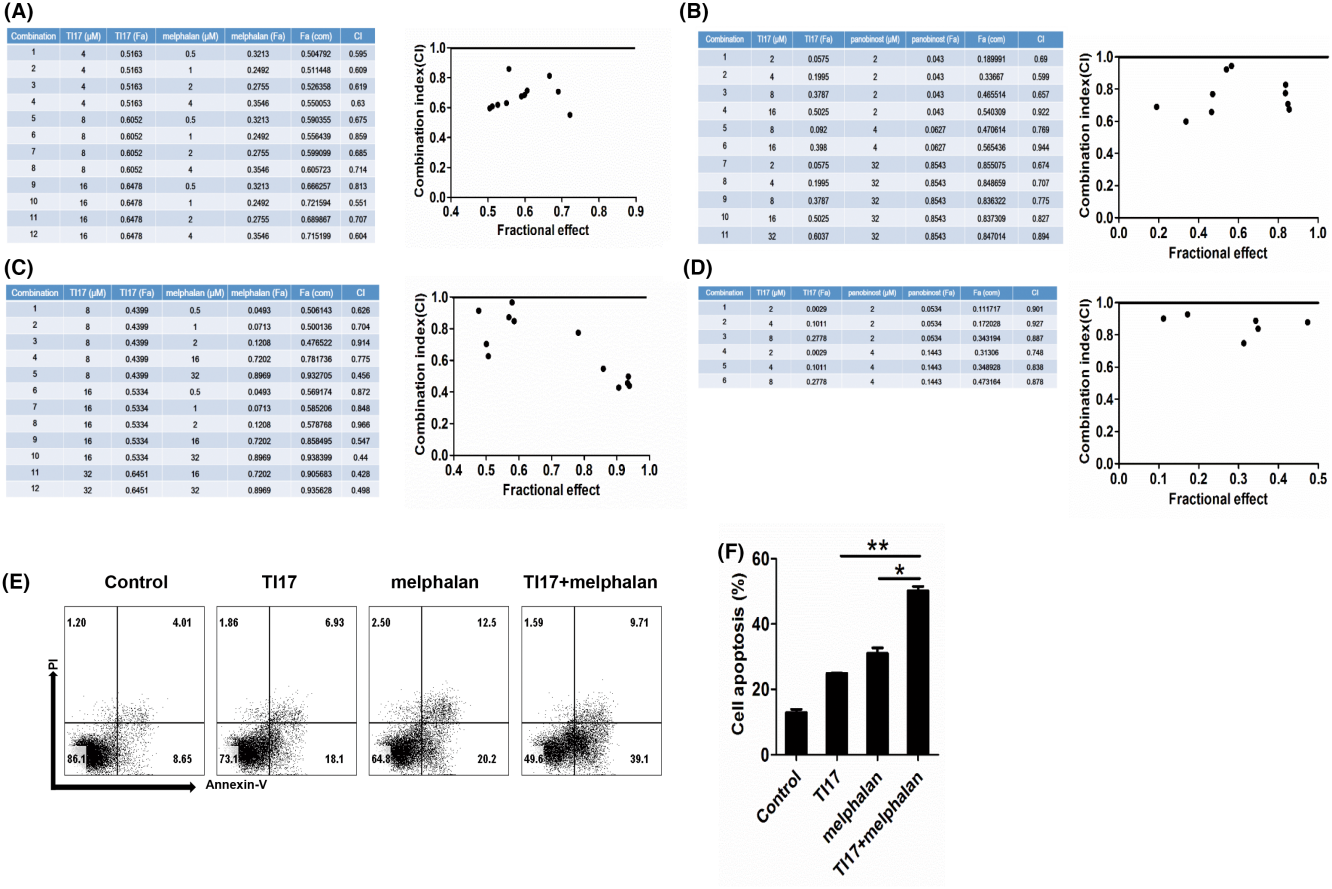
TI17 effects on MM cells in combination with MEL or HDAC inhibitor. ARP‐1 (A, B) and OCI‐MY5 (C, D) cells were combined treatment with TI17 and MEL or HDAC inhibitor panobinostat for 48 h, and cell viability was evaluated by using CCK8 kit, CI <1 indicates synergistic activity by using CalcuSyn software, Version 2.1. (E, F) ARP‐1 cells were treated with DMSO or TI17 (10 μM) and MEL (5 μM) for 48 h, and measured the cell apoptosis by Annexin V/PI double staining using flow cytometry. All results are expressed as mean ± SD of three independent experiments and *p ≤ 0.05, **p ≤ 0.01.

ARP‐1 and OCI‐MY5 cells were treated in combination with TI17 and MEL or HDAC inhibitor panobinostat. We observed that TI17 increased cytotoxicity of these agents in MM cells (Figure 8A–D). Meanwhile, we calculated the CI values by using CalcuSyn software. TI17 was synergistic with MEL (CI <1), or panobinostat (CI <1) against ARP‐1, as well as OCI‐MY5 cells. Importantly, we further found TI17 with MEL increased cell apoptosis in ARP‐1 cells by flow cytometry (Figure 8E,F), indicating that the synergistic cytotoxicity induced by combination with TI17 and MEL may due to apoptosis cell death.

## 4 DISCUSSION

Trip13 has a great amplification in high‐risk MM patients.^22^ Moreover, our previous studies have shown that knockdown Trip13 could against the growth of MM both in vivo and in vitro,^23^ suggesting Trip13 may represent an anti‐MM target and inhibition of Trip13 could be a promising therapeutic strategy for MM. In the present study, we have identified a novel small molecular inhibitor, TI17, which can impair DSBs repair by directly binding to Trip13, displays high potent anti‐MM activity in both in vitro and in vivo study.

We first found that TI17 was identified to target Trip13 via using the ligand observed T1ρ nuclear magnetic resonance (NMR) analysis. Furthermore, we observed TI17 could specifically bound endogenous Trip13 derived from MM cells and the proliferative inhibitions of TI17 was increased in Trip13‐overexpressed MM cells. Additionally, inhibition of ATPase activity after TI17 treatment further suggested that TI17 directly targets Trip13.

Through the study, we have demonstrated that TI17 treatment inhibits the growth of a large amount of MM cells in vitro. And we also observed that TI17 could trigger MM cell lines growth inhibition when MM cells were cultured with BMSC and cytokines IL‐6 and IGF‐1. These findings further indicate that as a small molecular inhibitor target trip13, TI17 has obvious anti‐MM activity in vitro.

The drugs of DNA damage and mitotic spindle damage always remain the main stream in the therapy of multiple cancer.^31^ Interestingly, our date indicated that TI17 induced accumulation of MM cells at the G0/G1 phase, suggesting that TI17 triggers DNA damage responses via inducing MM cells cycle arrest. Apoptosis could be triggered by endogenously or exogenously via death signal pathways.^32^ We observed that TI17 induced apoptosis of MM cells and primary CD138^+^ MM cells, while no obvious apoptosis on normal cells. Cleavage of caspase‐3, caspase‐8, and caspase‐9 was further confirming its high potent apoptosis.

**FIGURE 9:**
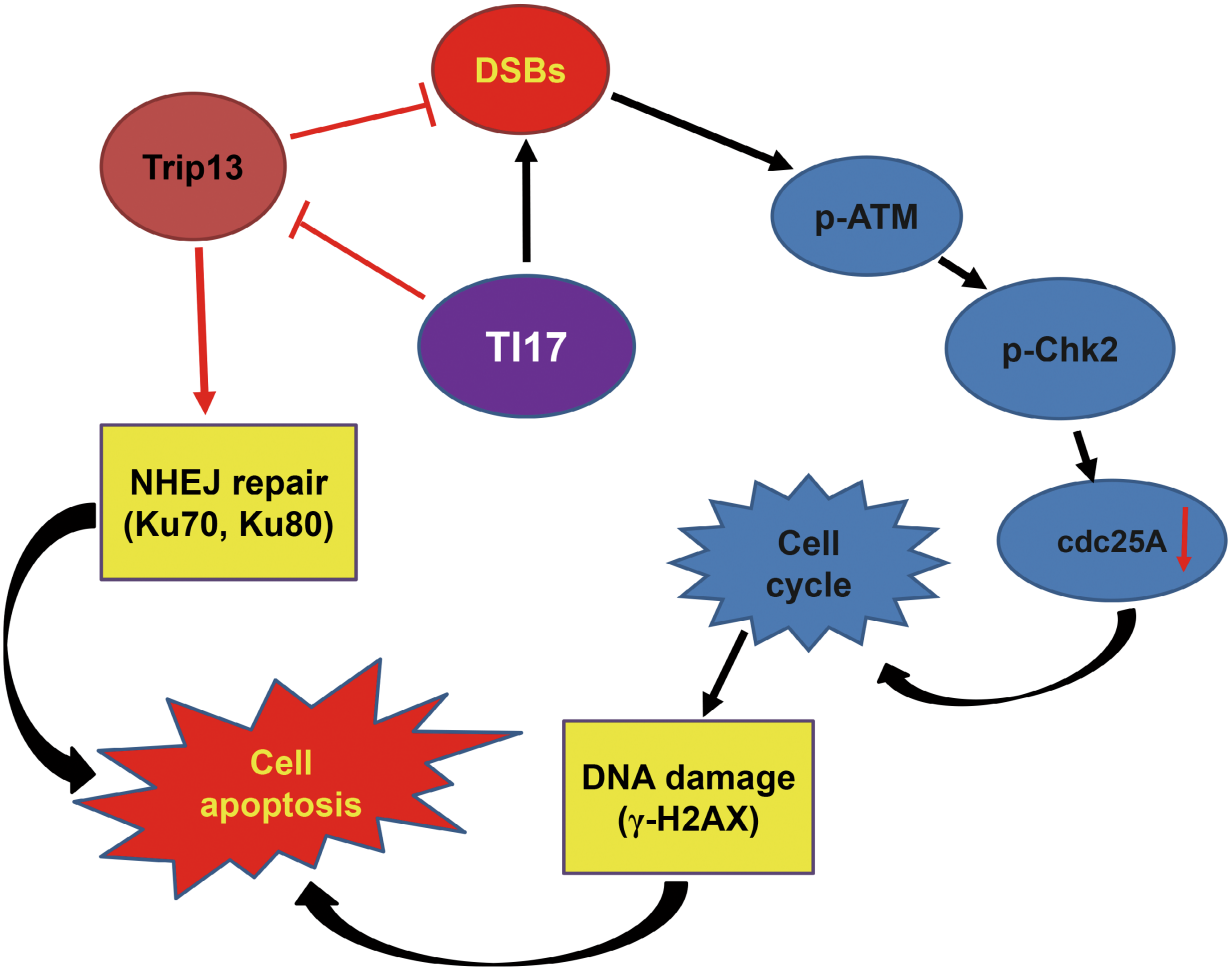
The molecular mechanism of TI17 effects on MM.

Trip13, belongs to the AAA family of chaperone proteins that involves in the assembly or disassembly of protein complexes.^33^ Ku is a DNA repair protein that is essential for binding to broken DNA and recruiting other proteins to promote the repair of DSBs.^34^ NHEJ is the primary pathway for DSBs repair in mammalian cells and is activated when DSBs ends were recognized by the Ku (Ku70 and Ku80) heterodimer.^35^ Consistently, we also observed the endogenous binding of Trip13 to Ku70 and Ku80 in MM cells by western blot of bead‐bound proteins, further strongly indicate that Trip13 plays an important role in cell survival by enhance NHEJ repair. Additionally, we found the expression of Ku70 and Ku80 in MM cells were significantly decrease after treatment with TI17, indicating that TI17 could decrease the DNA end‐binding protein Ku70/Ku80 and show an inability to repair DNA damage. As histone H2AX is phosphorylated at the Ser‐139 (γ‐H2AX) in response to DSBs, and DNA damage is prevalent in MM, evidenced by the presence of γ‐H2AX.^36^, ^37^ Furthermore, the absence of γ‐H2AX indicates efficient DSB‐repair, while higher γ‐H2AX performs impaired DSB‐repair.^38^ Our results indicated that treatment with TI17 increase the phosphorylation of H2AX and the presence of γ‐H2AX foci in MM cells. ATM plays a key role in regulating cellular response to DSBs, and its activation can lead to phosphorylation of many downstream targets that mediate DNA damage response pathways.^30^, ^39^ Importantly, we found that TI17 could phosphorylation of ATM by DSBs trigger, and phosphorylation of its downstream Chk2, followed by cdc25A decrease. Taken together, TI17 not only impaired the repair of DSBs, but also induced DNA damage responses in MM (Figure 9). Nonetheless, the detail molecular mechanism of TI17 directly targets to Trip13 effect on MM still needs to our further research.

In order to further observe the anti‐MM efficacy of TI17, we next developed tumor xenograft models in vivo. Our in vivo study indicated that TI17 treatment obviously inhibited the tumor growth, while without significant general toxicity. Meanwhile, Immunohistochemical staining of harvest tumors confirmed that TI17 inhibited the proliferation of MM cells and promoted their apoptosis. More importantly, the increase of γ‐H2AX expression in tumor further support TI17‐induced DNA damage responses. Therefore, TI17 is a well‐tolerated agent with potential therapeutic effect in MM.

As MM patients often occurs drug recurrence and poor prognosis, combinational therapy is needed after the initial treatment to increase the therapeutic effect and improve patient outcomes in MM.^40^, ^41^ CCK8 kit and flow cytometry analysis showed that TI17 in combination with MEL or panobinostat induced synergistic cytotoxicity in MM cells. Therefore, these data suggest that TI17 may have a promising application in clinical treatment.

Our studies showed that, TI17, a novel small molecular, effectively exert antitumor activity on MM by impairing Trip13 function of DSBs repair and enhancing DNA damage responses via inhibition of MM cells proliferation, accumulation of MM cells at G0/G1 phase, induction of MM cells apoptosis, and retardation of tumor growth in vivo. Collectively, our present study provides a new and effective therapeutic strategy for treating MM, and suggests the promise of therapeutics targeting Trip13 to improve MM treatment.

## AUTHOR CONTRIBUTIONS

**Shuaikang Chang:** Data curation (equal); methodology (equal); writing – original draft (equal). **Wenqin Xiao:** Data curation (equal); methodology (equal); writing – original draft (equal). **Yongsheng Xie:** Data curation (equal). **Zhijian Xu:** Methodology (equal). **Bo Li:** Validation (supporting). **Guanli Wang:** Validation (supporting). **Ke Hu:** Software (supporting). **Yong Zhang:** Software (supporting). **Jinfeng Zhou:** Validation (supporting). **Dongliang Song:** Investigation (supporting). **Huabin Zhu:** Methodology (supporting). **Xiaosong Wu:** Investigation (equal). **Yumeng Lu:** Writing – review and editing (equal). **Jumei Shi:** Conceptualization (equal); writing – review and editing (equal). **Weiliang Zhu:** Conceptualization (equal); writing – review and editing (equal).

## FUNDING INFORMATION

This work was supported by the National Natural Science Foundation of China (grant numbers 82000220, 81971529, 82070224, 82170200, and 82170201) and National Science & Technology Major Project “Key New Drug Creation and Manufacturing Program”, China (grant number 2018ZX09711002). Natural Science Foundation of Shanghai, China (grant number 19ZR1467800), and Shanghai Sailing Program (grant number 20YF1439600).

## CONFLICT OF INTEREST STATEMENT

The authors declare no conflict of interest.

## ETHICS APPROVAL AND CONSENT TO PARTICIPATE

All animal‐related procedures were approved by the Animal Care and Use Committee of Tongji University (Shanghai, China) and the institutional review board of Shanghai Tenth People's Hospital (ID: SYXK 2018–0034). Primary studies were approved by the Shanghai Tenth People's Hospital Ethics Committee. Informed consent was obtained from all study participants.

## Supporting information


Figure S1.
Figure S2.
Figure S3.
Figure S4.

## Data Availability

Data Availability StatementThe datasets used and/or analyzed during the current study are available from the corresponding author on reasonable request.

## References

[1] Rajkumar SV , Dimopoulos MA , Palumbo A , et al. International myeloma working group updated criteria for the diagnosis of multiple myeloma. Lancet Oncol. 2014;15:e538‐e548. doi:10.1016/s1470-2045(14)70442-5 25439696

[2] Kumar SK , Lee JH , Lahuerta JJ , et al. Risk of progression and survival in multiple myeloma relapsing after therapy with IMiDs and bortezomib: a multicenter international myeloma working group study. Leukemia. 2012;26:149‐157. doi:10.1038/leu.2011.196 21799510 PMC4109061

[3] Kumar SK , Rajkumar SV , Dispenzieri A , et al. Improved survival in multiple myeloma and the impact of novel therapies. Blood. 2008;111:2516‐2520. doi:10.1182/blood-2007-10-116129 17975015 PMC2254544

[4] Siegel DS , Schiller GJ , Samaras C , et al. Pomalidomide, dexamethasone, and daratumumab in relapsed refractory multiple myeloma after lenalidomide treatment. Leukemia. 2020;34:3286‐3297. doi:10.1038/s41375-020-0813-1 32376855 PMC7685974

[5] Lendvai N , Yee AJ , Tsakos I , et al. Phase IB study of cabozantinib in patients with relapsed and/or refractory multiple myeloma. Blood. 2016;127:2355‐2356. doi:10.1182/blood-2016-01-694786 27020089 PMC5003505

[6] Jackson SP , Bartek J . The DNA‐damage response in human biology and disease. Nature. 2009;461:1071‐1078. doi:10.1038/nature08467 19847258 PMC2906700

[7] Chapman JR , Taylor MR , Boulton SJ . Playing the end game: DNA double‐strand break repair pathway choice. Mol Cell. 2012;47:497‐510. doi:10.1016/j.molcel.2012.07.029 22920291

[8] Sartori AA , Lukas C , Coates J , et al. Human CtIP promotes DNA end resection. Nature. 2007;450:509‐514. doi:10.1038/nature06337 17965729 PMC2409435

[9] Lieber MR . The mechanism of human nonhomologous DNA end joining. J Biol Chem. 2008;283:1‐5. doi:10.1074/jbc.R700039200 17999957

[10] San‐Segundo PA , Roeder GS . Pch2 links chromatin silencing to meiotic checkpoint control. Cell. 1999;97:313‐324. 10319812 10.1016/s0092-8674(00)80741-2

[11] Bhalla N , Dernburg AF . A conserved checkpoint monitors meiotic chromosome synapsis in caenorhabditis elegans. Science. 2005;310:1683‐1686. doi:10.1126/science.1117468 16339446

[12] Joyce EF , McKim KS . Drosophila PCH2 is required for a pachytene checkpoint that monitors double‐strand‐break‐independent events leading to meiotic crossover formation. Genetics. 2009;181:39‐51. doi:10.1534/genetics.108.093112 18957704 PMC2621188

[13] Kang JU , Koo SH , Kwon KC , Park JW , Kim JM . Gain at chromosomal region 5p15.33, containing TERT, is the most frequent genetic event in early stages of non‐small cell lung cancer. Cancer Genet Cytogenet. 2008;182:1‐11. doi:10.1016/j.cancergencyto.2007.12.004 18328944

[14] Cai W , Ni W , Jin Y , Li Y . TRIP13 promotes lung cancer cell growth and metastasis through AKT/mTORC1/c‐Myc signaling. Cancer Biomark. 2021;30:237‐248. doi:10.3233/CBM-200039 33136091 PMC12499982

[15] Wang K , Sturt‐Gillespie B , Hittle JC , et al. Thyroid hormone receptor interacting protein 13 (TRIP13) AAA‐ATPase is a novel mitotic checkpoint‐silencing protein. J Biol Chem. 2014;289:23928‐23937. doi:10.1074/jbc.M114.585315 25012665 PMC4156041

[16] Li XC , Schimenti JC . Mouse pachytene checkpoint 2 (trip13) is required for completing meiotic recombination but not synapsis. PLoS Genet. 2007;3:e130. doi:10.1371/journal.pgen.0030130 17696610 PMC1941754

[17] Roig I , Dowdle JA , Toth A , de Rooij DG , Jasin M , Keeney S . Mouse TRIP13/PCH2 is required for recombination and normal higher‐order chromosome structure during meiosis. PLoS Genetics. 2010;6:e1001062. doi:10.1371/journal.pgen.1001062 20711356 PMC2920839

[18] Li XC , Bolcun‐Filas E , Schimenti JC . Genetic evidence that synaptonemal complex axial elements govern recombination pathway choice in mice. Genetics. 2011;189:71‐82. doi:10.1534/genetics.111.130674 21750255 PMC3176111

[19] Larkin SE , Holmes S , Cree IA , et al. Identification of markers of prostate cancer progression using candidate gene expression. Br J Cancer. 2012;106:157‐165. doi:10.1038/bjc.2011.490 22075945 PMC3251845

[20] Banerjee R , Russo N , Liu M , et al. TRIP13 promotes error‐prone nonhomologous end joining and induces chemoresistance in head and neck cancer. Nat Commun. 2014;5:4527. doi:10.1038/ncomms5527 25078033 PMC4130352

[21] Zhou W , Yang Y , Xia J , et al. NEK2 induces drug resistance mainly through activation of efflux drug pumps and is associated with poor prognosis in myeloma and other cancers. Cancer Cell. 2013;23:48‐62. doi:10.1016/j.ccr.2012.12.001 23328480 PMC3954609

[22] Shaughnessy JD Jr , Zhan F , Burington BE , et al. A validated gene expression model of high‐risk multiple myeloma is defined by deregulated expression of genes mapping to chromosome 1. Blood. 2007;109:2276‐2284. doi:10.1182/blood-2006-07-038430 17105813

[23] Tao Y , Yang G , Yang H , et al. TRIP13 impairs mitotic checkpoint surveillance and is associated with poor prognosis in multiple myeloma. Oncotarget. 2017;8:26718‐26731. doi:10.18632/oncotarget.14957 28157697 PMC5432292

[24] Xiao W , Xu Z , Chang S , et al. Rafoxanide, an organohalogen drug, triggers apoptosis and cell cycle arrest in multiple myeloma by enhancing DNA damage responses and suppressing the p38 MAPK pathway. Cancer Letters. 2019;444:45‐59. doi:10.1016/j.canlet.2018.12.014 30583070

[25] Wang Y , Huang J , Li B , et al. A small‐molecule inhibitor targeting TRIP13 suppresses multiple myeloma progression. Cancer Res. 2020;80:536‐548. doi:10.1158/0008-5472.CAN-18-3987 31732653

[26] Maurizio E , Wiśniewski JR , Ciani Y , et al. Translating proteomic into functional data: an high mobility group A1 (HMGA1) proteomic signature has prognostic value in breast cancer. Mol Cell Proteomics. 2016;15:109‐123. doi:10.1074/mcp.M115.050401 26527623 PMC4762532

[27] Carter SL , Eklund AC , Kohane IS , Harris LN , Szallasi Z . A signature of chromosomal instability inferred from gene expression profiles predicts clinical outcome in multiple human cancers. Nat Genet. 2006;38:1043‐1048. doi:10.1038/ng1861 16921376

[28] Li AY , Boo LM , Wang SY , et al. Suppression of nonhomologous end joining repair by overexpression of HMGA2. Cancer Res. 2009;69:5699‐5706. doi:10.1158/0008-5472.CAN-08-4833 19549901 PMC2737594

[29] Keith CT , Schreiber SL . PIK‐related kinases: DNA repair, recombination, and cell cycle checkpoints. Science. 1995;270:50‐51. 7569949 10.1126/science.270.5233.50

[30] Kastan MB , Bartek J . Cell‐cycle checkpoints and cancer. Nature. 2004;432:316‐323. doi:10.1038/nature03097 15549093

[31] Visconti R , Della Monica R , Grieco D . Cell cycle checkpoint in cancer: a therapeutically targetable double‐edged sword. J Exp Clin Cancer Res. 2016;35:153. doi:10.1186/s13046-016-0433-9 27670139 PMC5037895

[32] Kiraz Y , Adan A , Kartal Yandim M , Baran Y . Major apoptotic mechanisms and genes involved in apoptosis. Tumour Biol. 2016;37:8471‐8486. doi:10.1007/s13277-016-5035-9 27059734

[33] Neuwald AF , Aravind L , Spouge JL , Koonin EV . AAA+: a class of chaperone‐like ATPases associated with the assembly, operation, and disassembly of protein complexes. Genome Res. 1999;9:27‐43. 9927482

[34] Featherstone C , Jackson SP . Ku, a DNA repair protein with multiple cellular functions? Mutat Res. 1999;434:3‐15. 10377944 10.1016/s0921-8777(99)00006-3

[35] Brown JS , Lukashchuk N , Sczaniecka‐Clift M , et al. Neddylation promotes ubiquitylation and release of Ku from DNA‐damage sites. Cell Rep. 2015;11:704‐714. doi:10.1016/j.celrep.2015.03.058 25921528 PMC4431666

[36] Walters DK , Wu X , Tschumper RC , et al. Evidence for ongoing DNA damage in multiple myeloma cells as revealed by constitutive phosphorylation of H2AX. Leukemia. 2011;25:1344‐1353. doi:10.1038/leu.2011.94 21566653 PMC3940337

[37] Cottini F , Hideshima T , Xu C , et al. Rescue of Hippo coactivator YAP1 triggers DNA damage‐induced apoptosis in hematological cancers. Nat Med. 2014;20:599‐606. doi:10.1038/nm.3562 24813251 PMC4057660

[38] Fernandez‐Capetillo O , Allis CD , Nussenzweig A . Phosphorylation of histone H2B at DNA double‐strand breaks. J Exp Med. 2004;199:1671‐1677. doi:10.1084/jem.20032247 15197225 PMC2212807

[39] Kurz EU , Lees‐Miller SP . DNA damage‐induced activation of ATM and ATM‐dependent signaling pathways. DNA Repair. 2004;3:889‐900. doi:10.1016/j.dnarep.2004.03.029 15279774

[40] Norris EJ , DeStephanis D , Tunquist B , Usmani S , Ganapathi R , Ganapathi M . Cytotoxic efficacy of filanesib and melphalan combination is governed by sequence of treatment in human myeloma cells. Blood Cancer J. 2016;6:e480. doi:10.1038/bcj.2016.92 27716742 PMC5098264

[41] Richardson PG , Hungria VTM , Yoon SS , et al. Panobinostat plus bortezomib and dexamethasone in previously treated multiple myeloma: outcomes by prior treatment. Blood. 2016;127:713‐721. doi:10.1182/blood-2015-09-665018 26631116 PMC4760132

