# Supplementary material for: TI17, a novel compound, exerts anti‐MM activity by impairing Trip13 function of DSBs repair and enhancing DNA damage
Source: Cancer Med. 2023 Nov 9;12(23):21321–34. doi: 10.1002/cam4.6706 (PMC10726904; doi:10.1002/cam4.6706)

**Supplementary figure 1:**

Trip13 was overexpressed in multiple myeloma by using western blot analysis.

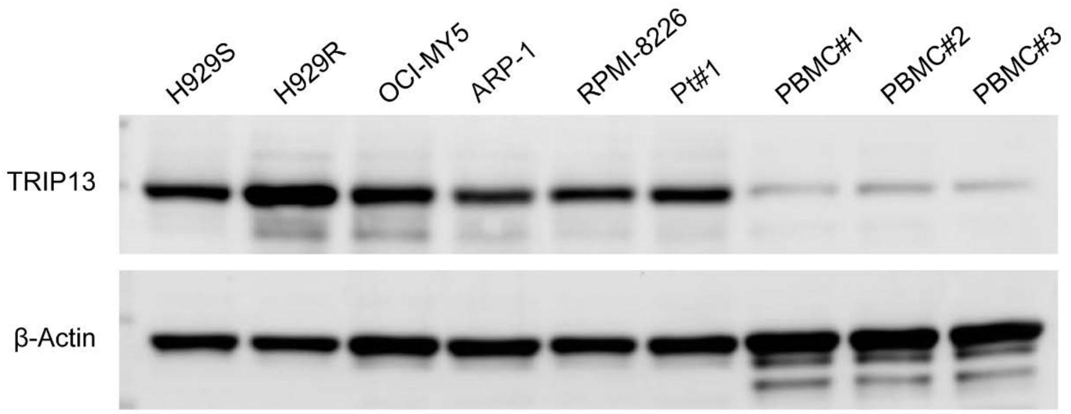

**Supplementary figure 2:**

Trip13 was overexpressed in other cancer cells and TI17 inhibits the viability of these cancer cells. **(A)** Western blot analysis of Trip13 in lung cancer A549 cells, prostate cancer PC-3 cells, nasopharyngeal cancer 5-8F cells, breast cancer MAD-MB-231 cells, liver cancer LM3 cells, and renal cancer A498 cells. **(B)** These cell lines were treated with DMSO or with TI17 for 48 h and cell viability was measured by CCK8 kit. **(C)** IC50 of these cell lines after DMSO or TI17 treatment was determined by using CalcuSyn software, Version 2.1.

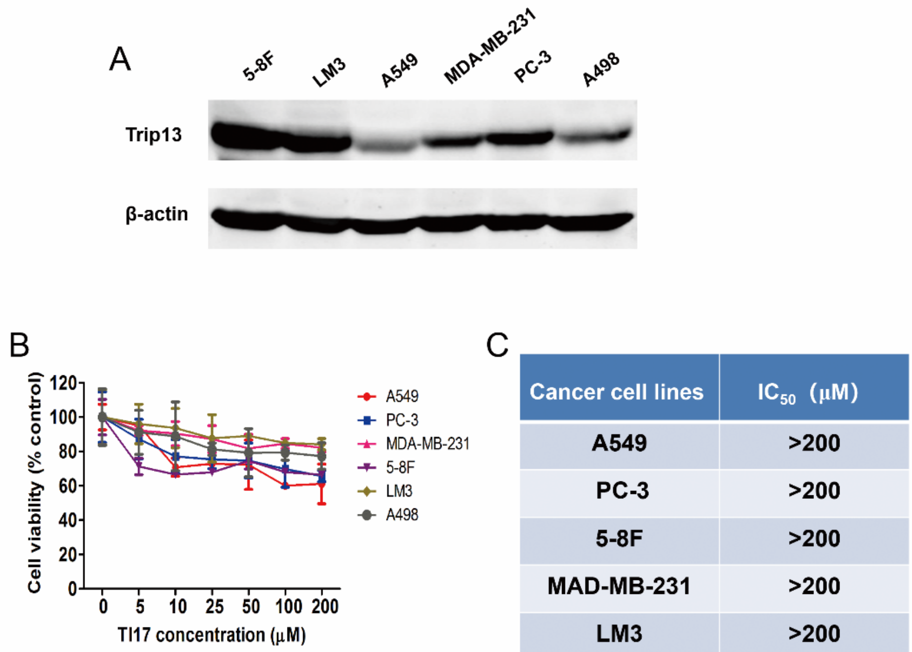


C

| Cancer cell lines | $IC_{50}$ (μM) |
|---|---|
| A549 | >200 |
| PC-3 | >200 |
| 5-8F | >200 |
| MAD-MB-231 | >200 |
| LM3 | >200 |
| A498 | >200 |

**Supplementary figure 3:**

TI17 induced apoptosis in MM cells in vitro. H929S **(A)** and H929R **(B)** cells were treated with DMSO or with TI17 for 48, and 72 h, and measured the cell apoptosis by Annexin V/PI double staining using flow cytometry.

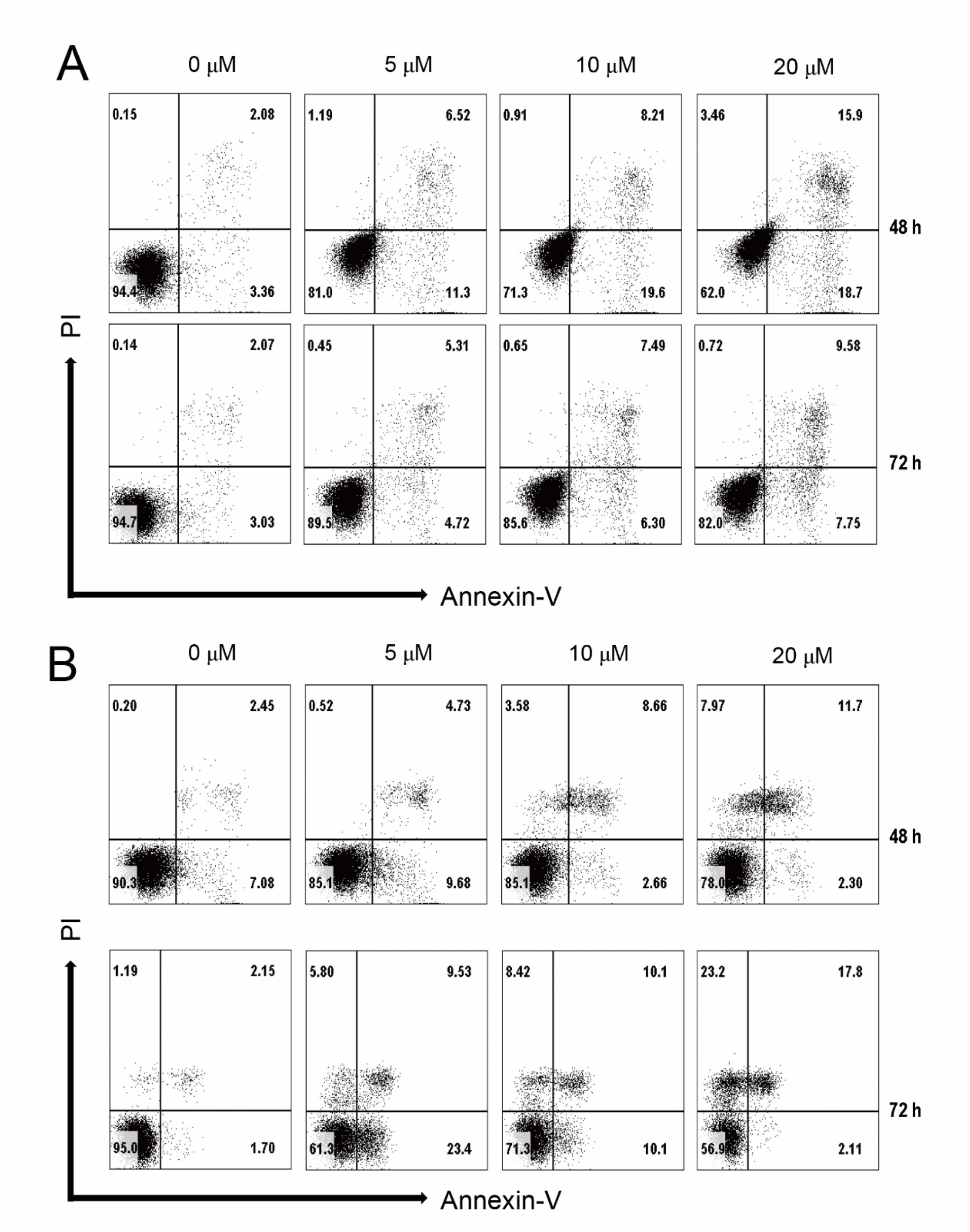

**Supplementary figure 4:**

HE staining of major organs for detected the histological change after TI17 treatment.

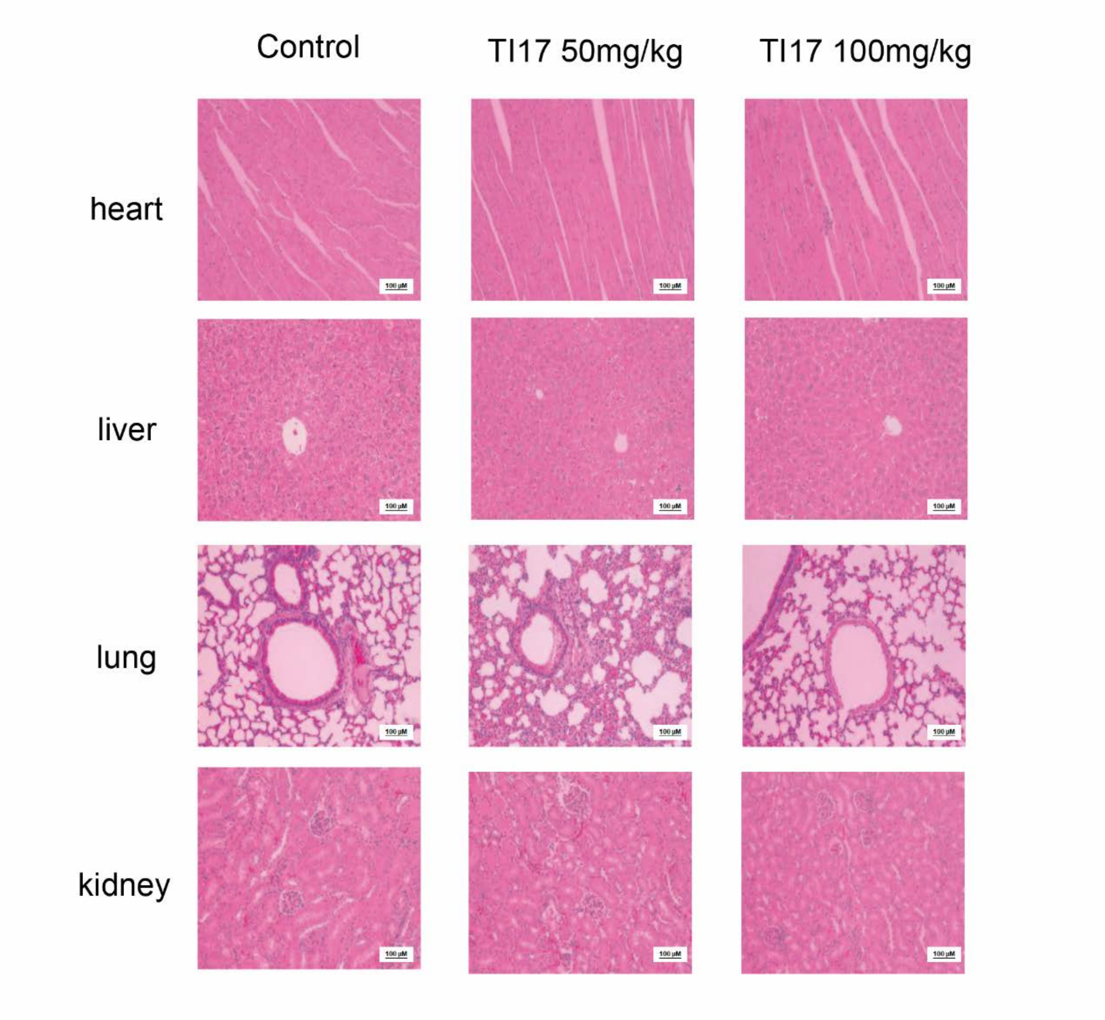

Supplement: Supplementary file 1 — Figure S1. Figure S2. Figure S3. Figure S4. [file CAM4-12-21321-s001.pdf]
